# Molecular detection of antimicrobial resistance in local isolates of Staphylococcus epidermidis from urinary tract infections in Faisalabad region of Pakistan

**DOI:** 10.17179/excli2015-294

**Published:** 2015-06-08

**Authors:** Anam Farid, Iram Naz, Asma Ashraf, Aamir Ali, Asad-ur Rehman, Yasra Sarwar, Abdul Haque

**Affiliations:** 1 Institute of Industrial Biotechnology, Government College University, Lahore; 2 Government College University, Faisalabad; 3 National Institute for Biotechnology and Genetic Engineering (NIBGE), Faisalabad; 4 Madina Teaching Hospital, The University of Faisalabad, Faisalabad, Pakistan

**Keywords:** Staphylococcus epidermidis, drug resistance, UTIs

## Abstract

*Staphylococci* are one of the foremost causes of urinary tract infections (UTIs) in humans. The emergence of multiple drug resistance (MDR) among *Staphylococci* poses serious challenges in antimicrobial therapy for UTIs. Most work has been done on *S*. aureus while coagulase negative *Staphylococci* (mainly *S. epidermidis*) are often neglected. This study was conducted to establish a baseline profile of drug resistance in local *S. epidermidis* isolates from UTIs. Eighty urine samples were collected from suspected UTIs cases and screened for *S. epidermidis*. Twenty isolates were suspected as *S. epidermidis* based on colony morphology and Gram staining. Molecular detection by polymerase chain reaction (PCR) confirmed 13 isolates as *S. epidermidis*. Using disc diffusion method, phenotypic drug resistance of the isolates was observed towards erythromycin (100 %), gentamycin, azithromycin and tetracycline (92.3 %), ampicillin and oxytetracyclin (84.6 %), amikacin and srteptomycin (76.9 %), methicillin (69.2 %), cephradine, cefaclor and cefazolin (53.8 %) and vancomycin (15.3 %). Eighteen most commonly reported genes responsible for conferring resistance towards these drugs were targeted by PCR: among these *tetM* gene was found most prevalent (46.1 %) followed by *tetK* (30.7 %), *aac(6')/aph(2”)* (30.7 %), *aacA-aphD* (23 %), *ermA* (23 %), *blaZ* (23 %), *mecA* (23 %) *blaTEM-1* (23 %), *MeccA* (23 %) and *mecA* (15.3 %). No gene fragment for vancomycin resistance was detected. The salient finding was that all *S. epidermidis* isolates were multiple drugs resistant as they showed resistance against at least three structurally different antimicrobial agents. It is concluded that in addition to the mostly used antimicrobial agent vancomycin, the cephalosporins including cephradine, cefaclor and cefazolin are also the drugs of choice against UTIs caused by *S. epidermidis*.

## Introduction

Urinary tract infections (UTIs) caused by pathogenic bacteria are considered as the second common category of infections in the body and around 8.1 million visits to health care are reported each year (Griebling, 2004[14]). The UTIs are often asymptomatic, although occasionally they produce distress in kidney transplant recipients, patients with an invasive genitourinary procedure, in pregnant women and in neutropenic patients (Gleckman, 1992[11]). Symptomatic UTIs can be either uncomplicated involving only urinary bladder or complicated leading to pyelonephritis or other metabolic and anatomical disorders. In patients with symptomatic infections presence of inflammatory cells and > 10^5^ bacteria per ml in freshly voided urine are reported (Lee and Neild, 2007[19]). There are several factors that affect the clinical manifestation of UTIs e.g. it depends on the severity of the infection, part of the urinary tract affected, the etiologic organism and patient's ability to mount strong immune response. Common symptoms are fever, urinary urgency, chills, dysuria and cloudy urine (Mori et al., 2007[25]). UTIs in children are more severe because they are more likely to damage the kidneys. Among children, poor urine control and bedwetting during the day are common signs (Lee and Neild, 2007[19]).

The risk of UTIs is more in females; one reason is the shorter urethra in women that allows bacteria quicker access to reach the bladder (Farshad et al., 2010[9]). Recurrent UTIs are common clinical problems and about 25 % of women with an acute UTI suffer a recurrence in 6 months, even when they are receiving a proper antibiotic therapy (Schilling et al., 2002[29]). Significant rates of morbidity and even mortality are observed in many cases of UTIs. It increases the risk of fetal mortality especially in weak elderly persons, pregnant women and pediatric patients (Martineau et al., 2000[23]). Urosepsis leading to death is also reported. Epidemiology of UTIs varies with several factors like gender, age, and the existence of genitourinary disorders (Griebling, 2004[14]).

Bacteria are considered as the most common cause of lower and upper urinary tract infections. Among bacterial infections, *Escherichia coli* is reported as the most common bacterium and it accounts for about 85 % of community-acquired and 50 % of hospital acquired urinary tract infections followed by *Staphylococcus *species that contribute up to 15 % (Vasudevan, 2014[34]).

*Staphylococcus epidermidis* is a coagulase negative *Staphylococci *and one of the most often reported *Staphylococcus* in nosocomial infections. It usually enters the urinary tract through urethra and resides on the skin or mucous membrane (Golding et al., 2012[12]). Recently, the *S. epidermidis* isolated from the hospital environment (catheter-related bloodstream infections and healthcare workers) was found resistant to more antibiotics than *S. epidermidis *colonizing healthy volunteers. It clearly suggests the *S. epidermidis* as potential cause of catheter related infections due to its antimicrobial resistance based adaptability to survive in the hospital environment (Cherifi et al., 2014[3]). To the best of our knowledge, there is no report earlier on molecular detection of antimicrobial resistance is *S. epidermidis* from this region.

## Materials and Methods

### Sample collection and bacterial isolation

Urine samples (no = 80) were aseptically collected from patients of all age groups and both sexes. The samples were collected from different laboratories and hospitals (Millat laboratory, PINUM hospital, Nusrat laboratory, Allied Hospital) of Faisalabad. Sterile leak resistant containers were used for collection and transportation of urine from hospitals to our laboratory and inoculated into 0.6 % nutrient agar in tryptic soya broth. The well-mixed unspun urine was streaked on nutrient ager plates using sterile swab and incubated overnight at 37 °C. The agar plates were observed for the presence of *Staphylococcus* colonies. The suspected isolates were sub-cultured on the same medium for obtaining pure cultures and processed for biochemical and molecular identification.

### Biochemical identification of isolates

The isolates suspected as *Staphylococcus *were processed for Gram-staining and observed under light microscope (Buck, 1982[2]). The coagulase test was performed (Skinner et al., 2009[30]) on glass slides to detect coagulase negative *Staphylococci*. Briefly, the isolates were cultured on nutrient agar plates and a suspension of colonies was made with saline. Two drops of the suspension were mixed with same volume of human blood plasma by sterilized plastic tip and the results regarding clumping or otherwise were noted within 10 seconds while gently rotating the glass slide in circular manner**.**

### Molecular identification of Staphylococcus epidermidis

Total genomic DNA from all isolates suspected as *Staphylococci *was extracted from the overnight culture at 37 °C in tryptic soy broth (TSB) using the phenol-chloroform method (Sambrook et al., 1989[28]). The integrity of DNA samples was checked by electrophoresis on 1 % agarose gel. Polymerase chain reaction (PCR) was performed for the confirmation of *S. epidermidis *isolates with SE-F and SE-R primers (Martineau et al., 2000[23]) targeting 124 base pairs *S. epidermidis* specific gene fragment. The 25 µL PCR reaction mixture including 5 µL of diluted template DNA was made up of 2.5 µL of 10X PCR buffer, 1.6 µL of 25mM MgCl_2_, 1.6 µL of dNTPs, 1.5 µL of each primer and 0.3 µL of *Taq* polymerase. The thermal cycler conditions were 95 °C for 3 min (initial denaturation), followed by 35 cycles of 95 °C for 1 min, 60 °C for 40 sec, 72 °C for 50 sec and a final extension of 72 °C for 7 min.

### Antimicrobial susceptibility testing

After PCR confirmation of the isolates, antimicrobial susceptibility testing was performed by disc diffusion method against six different antimicrobial groups, using discs of thirteen representative antimicrobials: amikacin (30 μg), ampicillin (10 μg), cefradine (30 μg), cefaclor (30 μg), cefazolin (30 μg), gentamicin (10 μg), streptomycin (10 μg), vancomycin (30 μg), azithromycin (15 μg), erythromycin (15 μg), methicillin (10 μg), tetracycline (30 μg) and oxytetracycline (30 μg). The diameter of zone of inhibition, if present was measured and results for all antimicrobials used were interpreted according to the guidelines of Clinical And Laboratory Standard Institute (CLSI, 2012[5]).

### Molecular detection of antimicrobial drug resistance genes

PCR was used to amplify 18 most commonly targeted drug resistance gene fragments in all *S. epidermidis* isolates as described by previous studies. For aminoglycosides (amikacin, gentamicin, streptomycin) the resistance genes (*aac*(*6'*)*/aph(2”*) (Kozitskaya et al., 2004[18]) and *aacA-aphD* (Strommenger et al., 2003[31]) were targeted while *bla*_TEM-1 _resistance gene fragment was targeted (Yan et al., 2000[38]) for cephalosporins (cefaclor, cefazolin and cephradine). For macrolides (erythromycin and azithromycin) the *ermA* and *ermC* resistance gene fragments were targeted (Strommenger et al., 2003[31]). For tetracyclines (tetracycline and oxytetracycline) the *tetM* and *tetK* gene fragments were targeted (Strommenger et al., 2003[31]). For beta-lactams (ampicillin and methicillin), the targeted gene fragments were *blaZ* (EI Zubeir et al., 2007[8])*, mecA* (EI Zubeir et al., 2007[8]), *meccA* (Del Vecchio et al., 1995[6])*, *and *MeccA* (Vannuffel et al., 1995[33]). Seven different resistance gene fragments including *vanR*, *vanS*, *vanH*, *vanA, vanX, vanY* and *vanZ* were targeted (Dezfulian et al., 2012[7]) for detection of vancomycin (glycopeptides) resistance genes. The sequences of primers along with sizes of amplicons are mentioned in Table 1(Tab. 1).

## Results

**Figure 1:**
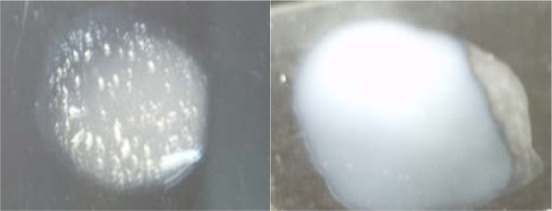
Coagulase test for *Staphylococci*. Control test (left) showing clumping when *Staphylococcus aureus* suspension reacted with human plasma while the reaction test (right) with coagulase negative *Staphylococci* showing no clumping

Out of 80 collected samples, 20 were suspected as *Staphylococci* based on colony morphology and Gram staining (positive). All these isolates were found coagulase negative as no clumping of human plasma was observed with them while the positive control *Staphylococcus aureus *showed clear clumping (Figure 1(Fig. 1)). The PCR confirmed 13 isolates as *S. epidermidis* as amplified product of 124 base pairs was observed in each case with no amplification in negative control.

**Figure 2:**
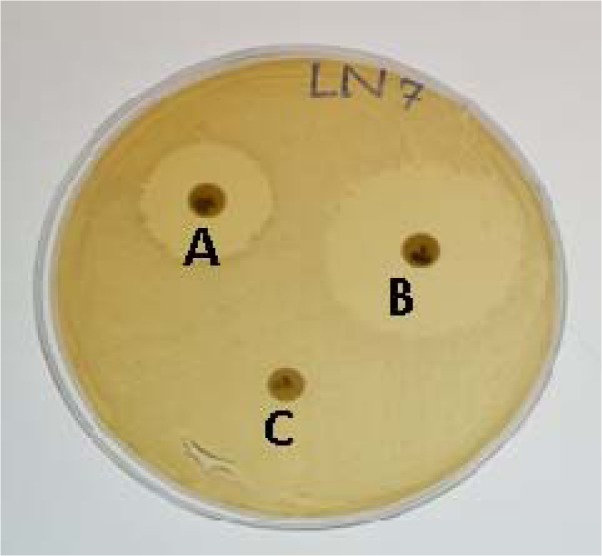
Antimicrobial drug resistance *Staphylococci *on Muller Hinton agar plates. A: Bacteria showing intermediate resistance against the antimicrobial. B: Bacteria showing complete susceptibility against the antimicrobial as indicative by the clear zone of inhibition. C: Bacteria showing resistance as no zone of inhibition.

The results of antimicrobial sensitivity testing by disc diffusion method for the 13 *S. epidermidis *isolates showed that all isolates have phenotypic resistance to at least three antimicrobial drugs (belonging to three different groups) so all the isolates were considered as multiple drug resistant (MDR) isolates (Zhao et al., 2005[40]). With disc diffusion method, all 13 (100 %) isolates showed resistance to erythromycin, whereas 12 (92.3 %) were resistant to gentamycin, azithromycin, and tetracycline. Eleven isolates (84.6 %) were resistant to ampicillin and oxytetracyclin, 10 (76.9 %) were found resistant to amikacin and streptomycin, 9 isolates (69.2 %) were resistant to methicillin and only 7 isolates (53.8 %) were resistant to cephradine, cefaclor and cefazolin while only two isolates (15.3 %) were found phenotypically resistant to vancomycin (Figure 2(Fig. 2)) but no gene for vancomycin resistance was detected.

**Figure 3:**
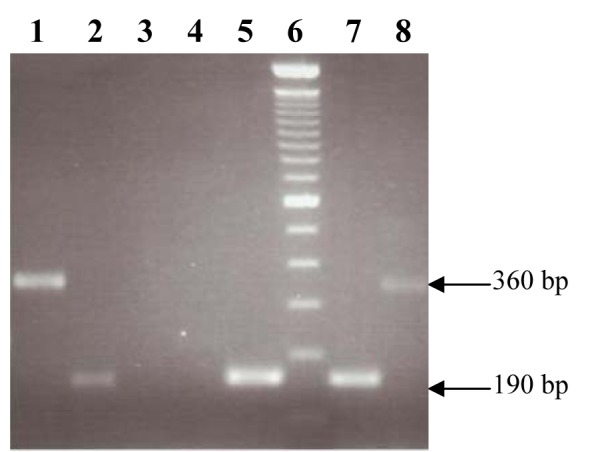
Amplification of drug resistance genes. Lanes1 and 8: Amplified product of 360 bp of *tetK* gene fragment. Lanes 2, 5 and 7: Amplified product of 190 bp of *erm(A)*. Lane 6: 100 bp GeneRuler (invitrogen).

The PCR based detection of different antimicrobial resistance genes showed the presence of *aacA-aphD* gene in 3 isolates (23 %)*, erm(A) *gene fragment in 3 isolates (23 %), *tetK* gene fragment (360 bp) in 4 isolates (30.7 %) and *tetM* in 6 (46.1 %) isolates. The *blaZ* gene which confers resistance to penicillin was detected in 3 isolates (23 %). No amplification was observed with primer sets used for genes reported to confer resistance against vancomycin. In the duplex PCR for *MeccA* and *mecA*, responsible for conferring resistance to methicillin, the *MeccA *was amplified in 3 isolates (23 %) and *mecA* was amplified in 2 isolates (15.3 %) while in 3 phenotypically resistant isolates, no gene was detected for methicillin resistance. The gene fragment of *bla*_ TEM-1 _was amplified (962 bp) in 3 isolates (23 %) while the gene *aac(6')/aph(2”)* responsible to confer resistance to gentamicin was found (1184 bp) in 4 isolates (30.7 %). The representative amplified products are shown in Figure 3(Fig. 3). 

## Discussion

The urinary tract of humans is a multi-organ system comprises of kidneys, bladder, urethra and ureters. Urinary tract infections (UTIs) are usually caused by pathogenic microorganisms (viruses, bacteria, fungi or parasites). These are considered the second common category of infections in the body (Griebling, 2004[14]). The urosepsis, pyelonephritis, metabolic and anatomical disorders are observed in complicated infections of the urinary tract that lead to tissue injury. It usually enters in the urinary tract through urethra and resides on the skin or mucous membrane (Golding et al., 2012[12]). There are different groups of antimicrobial drugs that act on bacteria including *S. epidermidis* in different ways, as each antimicrobial drug has a specific mode of action (Neu, 1992[26]). 

Beta-lactam drugs including penicillins, ampicillins and oxacillins are considered to be a major group of antimicrobial drugs. Resistance to penicillins is mostly caused by the presence of ß-lactamases, but mutations in penicillin binding proteins (PBPs) of bacteria also have resulted in reduced affinity for ß-lactams (Brinas et al., 2005[1]). Methicillin and amplicillin are used as representatives of this group. Methicillin resistance in *Staphylococci* has been reported to be associated with the presence of PBPs encoded by the *MeccA* gene (Zapun et al., 2008[39]; Choi et al., 2003[4]). The high level of resistance was found against methicillin (69.2 %) in our isolates using disk diffusion method but the genes involved in conferring resistance to methicillin (*MeccA* and *mecA*), were identified in three and two isolates respectively. The possible reason for this difference in genotypic and phenotypic antimicrobial sensitivity patterns is the presence of other drug resistance genes which were not included in this study. Results about resistance to methicillin found in this study were higher than those reported (34.2 %) from Turkey (Hosgor et al., 2007[16]) and Europe (25 %) (Fluit et al., 2001[10]) but were comparable to those reported from USA (Marshall et al., 1998[22]).

The resistance against gentamicin was found in 12 (92.3 %) isolates in our study by disc diffusion method. The presence of aminoglycoside-modifying enzyme (AME) genes *aac*(6')/*aph*(2”) and *aacA*-*aphD* gene fragments was also checked. The gene *aac*(6')/*aph*(2”) responsible to confer resistance against gentamicin was amplified (1184 bp) in 4 isolates (30.7 %) and *aacA-aphD *was amplified (227 bp) in 3 isolate (23 %). In accordance with previous studies (Vanhoof et al., 1994[32]; Choi et al., 2003[4]), we also found the *aac(6')/aph(2'')* gene as the most prevalent AME gene in *Staphylococci.* Contrarily, a study from Japan reported less frequent detection of *aac(6')/aph(2'')* gene among clinical MRSA isolates (Ida et al., 2001[17]).

In case of erythromycin, all (13) isolates were found phenotypically resistant while PCR assay detection *erm*(*A*) gene fragment in only 3 isolates (23 %) while no amplification was observed for *erm*(*C*) gene fragment. Previously, the *erm*(*A*)gene was reported as more prevalent than the other erythromycin resistance genes in *S. aureus* while *erm*(*C*) gene is present more frequently in coagulase negative *Staphylococci* (Lim et al., 2002[20]). Erythromycin resistant might be due to the presence of *msrA *or *ermB *gene fragments as previously described (Weisblum, 1995[35]) which were not included in our study. The resistance against tetracycline and oxytetracyclin was phenotypically observed in 12 (92.3 %) and 11 (84.6 %) isolates respectively. Tetracycline resistance gene fragments *tetK* and *tetM *were targeted in this study and *tetK* was amplified in 3 (23 %) isolates while *tetM *gene fragment was amplified in 6 (46.1 %) isolates. However, we found neither of these genes in 4 (30.7 %) phenotypically resistant isolates which indicates the possibility of carrying some other tetracycline resistance genes (Roberts, 2005[27]) or harboring some other antimicrobial resistance phenomenon (Guardabassi et al., 2000[15]).

In this study, we found 2 (15.3 %) isolates resistant to vancomycin (phenotypically) by disc diffusion method but not a single isolate showed amplification in the multiplex PCR for the targeted seven gene fragments i.e. *vanR, vanS, vanH, vanA, vanX, vanY*and *vanZ. *One possible reason is that the most effective form of vancomycin resistance depends on a transposon containing seven genes, the products of which work together to sense the presence of vancomycin, shut down the normal pathway for bacterial cell wall synthesis, and generate a different type of cell wall. Therefore, the joining of these genes into a single transposon must have been a difficult evolutionary step. Moreover the bacteria may have some other alternative strategies to confer resistance against vancomycin. There are only a few cases reported all over the world about isolation of vancomycin resistant *S. aureus* (VRSA) from clinical specimens and among these isolates small number was community-acquired. The first case of community-acquired methicillin and vancomycin-resistant *S. aureus* was reported in Tehran, Iran. According to it in-vitro transfer of vancomycin resistance gene (*vanA*) from the source *Enterococci* to *S. aureus* isolates, it was suspected that there is a possibility of transformation of *vanA* gene from vancomycin resistant *Enterococci* (VRE) to *Staphylococci* species (Whitener et al., 2004[36]).

Increased resistance in bacteria particularly in methicillin resistant *S. aureus* (MRSA) to several antimicrobial agents has aggravated due to the over use of vancomycin as a first-line empirical therapy as well as in prophylaxis therapy, though the selection of vancomycin resistance and the potential transmission of resistance in species encourage restricted use of these glycopeptides (Maccanti and Bonadio, 1992[21]; Grayson, 1993[13]).

Another major group of antimicrobial drugs is cephalosporins. These drugs are very efficient and several generations are available in the market. First generation preparations (cefaclor, cefadroxil, cephazolin, cefradine, cephalexin, cephaloridine etc.) are generally active against gram-positive bacteria. We used three drugs i.e. cephazolin, cefradine and cefaclor. With disc diffusion method, 7 (53.8 %) isolates were observed to be resistant against these antimicrobials. While in a multiplex PCR assay *bla *TEM-1 gene involved in conferring resistance to cephalosporins was found in only 3 isolates (23 %). We conclude that vancomycin is the drug of choice against UTIs caused by Staphylococci and cephalosporins including cephradine, cefaclor and cefazolin are also still effective in this geographic region.

## Figures and Tables

**Table 1:**
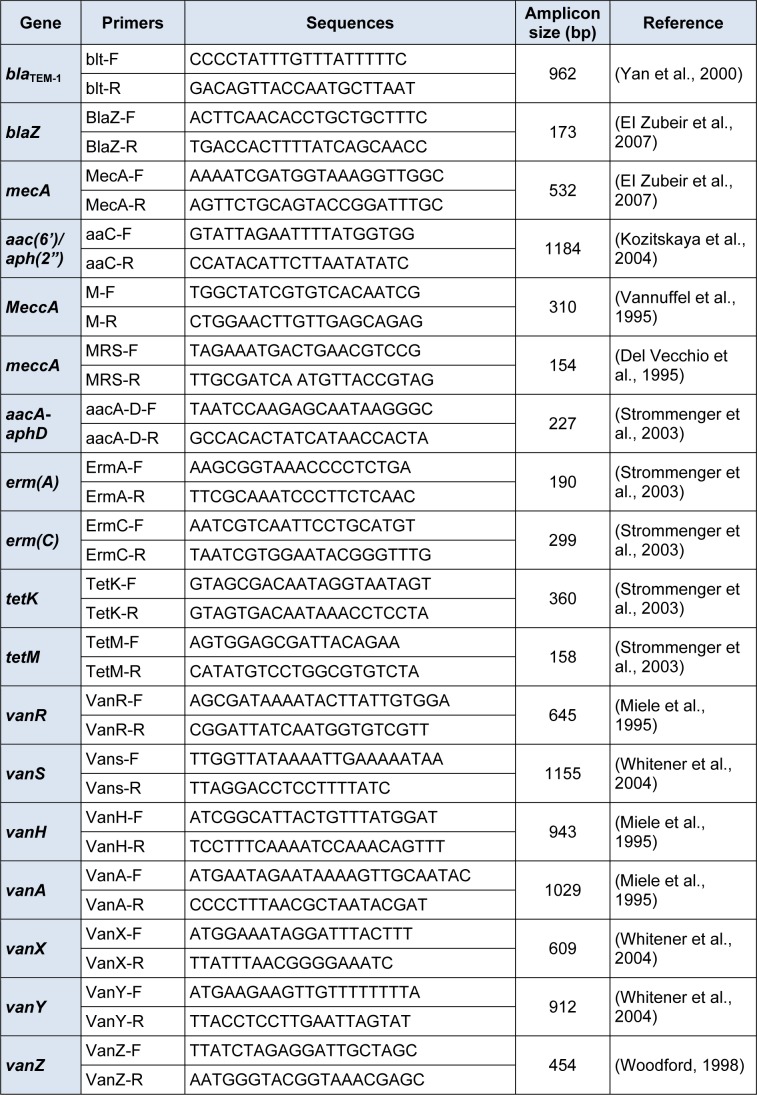
Drug resistance primers used in the study
